# Chromatin remodeling protein HELLS is critical for retinoblastoma tumor initiation and progression

**DOI:** 10.1038/s41389-020-0210-7

**Published:** 2020-02-18

**Authors:** Loredana Zocchi, Aditi Mehta, Stephanie C. Wu, Jie Wu, Yijun Gu, Jingtian Wang, Susie Suh, Robert C. Spitale, Claudia A. Benavente

**Affiliations:** 1 Department of Pharmaceutical Sciences, University of California, Irvine, CA 92697 USA; 2 Pediatric Hematology and Pediatric Oncology, Children’s Hospital of Orange County, Orange, CA 92868 USA; 3 Department of Graduate Medical Education, University of California, Irvine, CA 92697 USA; 4 Department of Developmental and Cell Biology, University of California, Irvine, CA 92697 USA; 5 Department of Biological Chemistry, University of California, Irvine, CA 92697 USA; 6 Chao Family Comprehensive Cancer Center, University of California, Irvine, CA 92697 USA; 7 Department of Molecular Biology and Biochemistry, University of California, Irvine, CA 92697 USA; 8 Gavin Herbert Eye Institute, Department of Ophthalmology, University of California, Irvine, CA 92697 USA

**Keywords:** Paediatric cancer, Cancer genetics, Epigenetics

## Abstract

Retinoblastoma is an aggressive childhood cancer of the developing retina that initiates by biallelic *RB1* gene inactivation. Tumor progression in retinoblastoma is driven by epigenetics, as retinoblastoma genomes are stable, but the mechanism(s) that drive these epigenetic changes remain unknown. Lymphoid-specific helicase (HELLS) protein is an epigenetic modifier directly regulated by the RB/E2F pathway. In this study, we used novel genetically engineered mouse models to investigate the role of HELLS during retinal development and tumorigenesis. Our results indicate that *Hells*-null retinal progenitor cells divide, undergo cell-fate specification, and give rise to fully laminated retinae with minor bipolar cells defects, but normal retinal function. Despite the apparent nonessential role of HELLS in retinal development, failure to transcriptionally repress *Hells* during retinal terminal differentiation due to retinoblastoma (RB) family loss significantly contributes to retinal tumorigenesis. Loss of HELLS drastically reduced ectopic division of differentiating cells in *Rb1/p107*-null retinae, significantly decreased the incidence of retinoblastoma, delayed tumor progression, and increased overall survival. Despite its role in heterochromatin formation, we found no evidence that *Hells* loss directly affected chromatin accessibility in the retina but functioned as transcriptional co-activator of E2F3, decreasing expression of cell cycle genes. We propose that HELLS is a critical downstream mediator of E2F-dependent ectopic proliferation in RB-null retinae. Together with the nontoxic effect of HELLS loss in the developing retina, our results suggest that HELLS and its downstream pathways could serve as potential therapeutic targets for retinoblastoma.

## Introduction

The RB pathway can directly regulate genes that control cell cycle exit but can also regulate the expression of genes that control cell-fate specification and differentiation through mechanisms, including chromatin remodeling and epigenetic control^1^. In the developing retina, the RB pathway is critical in development and tumorigenesis. Genetic ablation of RB is characterized by alterations in ganglion cell, bipolar, and rod photoreceptor terminal differentiation that results in cell death of these retinal cells and disruption of horizontal cell synaptogenesis^2,3^. Further, almost all retinoblastomas have *RB1* gene inactivation^4,5^. While genetic alterations in the *RB1* gene are required for tumor initiation, retinoblastoma tumors have stable genomes, with tumor progression occurring through epigenetic dysregulation of several cancer pathways^6^.

*HELLS* (helicase, lymphoid specific; also known as LSH, ICF4, PASG, and SMARCA6), a gene transcriptionally controlled by the RB/E2F pathway^7–9^, encodes a chromatin remodeling protein thought to be responsible for the epigenetic changes seen in retinoblastoma and required for tumor survival^10^. HELLS is a SWI/SNF-related matrix-associated regulator of chromatin that contributes to global genome methylation^11^. HELLS remodels chromatin to render DNA accessible to DNA methyltransferase enzymes Dnmt3a or Dnmt3b, supporting de novo DNA methylation and stable gene silencing during cellular differentiation^12,13^. Binding of HELLS to DNMT1, HDAC1, and HDAC2 has also been reported^14^. HELLS plays an important role in normal development as HELLS knockout mice display perinatal lethality^15^. Furthermore, HELLS mutant mice exhibit signs of growth retardation, premature aging, and impaired neural stem/progenitor cells self-renewal and maintenance during development^16,17^. Thus, consideration of HELLS as a therapeutic target in the treatment of cancers requires careful consideration of the potential toxicities that could arise in tissues exposed to treatment.

**Fig. 1:**
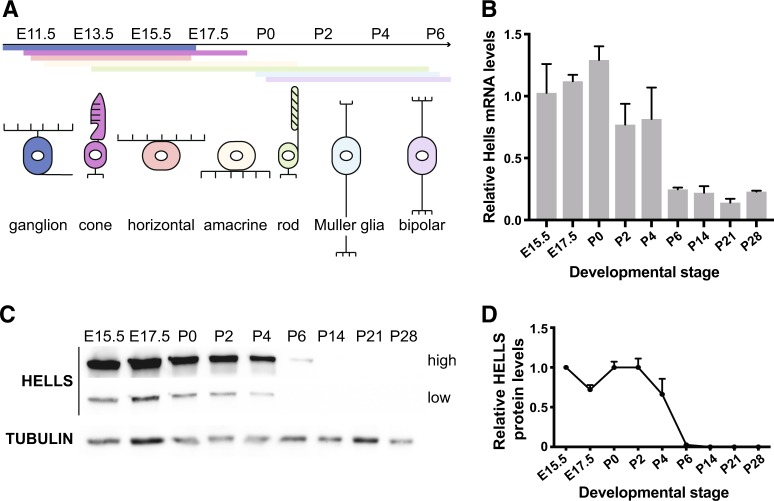
HELLS is repressed during the late stages of retinal development. **a** Illustration of the developmental stages and specification timing of the seven major retinal cell types: retinal ganglion cells, amacrine, horizontal, bipolar, Müller, cone, and rod photoreceptors. **b** Time course of *Hells* mRNA expression in the developing retina in wild-type mice. Levels of *Hells* mRNA were measured by RT-qPCR and normalized to the levels at E15.5 taken as 1 (*n* = 3). Mean ± SD. **c** Representative western blot analysis of HELLS protein levels with high and low exposure. Tubulin was used as a loading control. **d** Quantification of the relative HELLS protein levels on western blots were E15.5 was taken as 1 (*n* = 3). Mean ± SD.

The vertebrate neural retina is organized with remarkable precision into laminar structure formed by multiple types of neurons and glial cells. In mouse retina, the seven major retinal cell types (rod and cone photoreceptors; horizontal, amacrine, and bipolar interneurons; Müller glia; and ganglion cells) are differentiated from a common population of multipotent retinal progenitor cells (RPCs) that appear between embryonic day 11 (E11) and postnatal day 10 (P10) in a conserved temporal order (Fig. 1a)^18,19^. The process of self-renewal and differentiation required for RPCs to differentiate into the distinct neural lineages is regulated by various molecular mechanisms, including transcriptional and post-transcriptional regulation. Epigenetic regulation plays a fundamental role in the maintenance of cell identity as well as the stepwise control toward cellular differentiation. Chromatin states of RPCs change gradually during this process and alterations of epigenetic markers during retinal development have been linked to several ocular diseases, including retinoblastoma^6,10,20–23^. Thus, understanding the molecular mechanism and identifying key chromatin factors that regulate retinal stem cell maintenance and neurogenesis is critical for understanding normal retinal development and discovering molecular pathways that could be targeted for cancer therapy and intraocular delivery.

To investigate the role of HELLS in normal retinal development and tumorigenesis, we generated a series of genetically engineered mouse models. Using a *Hells* conditional knockout mouse model driven by *Chx10-*cre recombinase, our results demonstrate that *Hells*-null RPCs divide, undergo cell-fate specification, and give rise to normally laminated retinae with all seven major retinal cell types present in normal ratios. While minor synaptogenesis defects were observed in a subset of bipolar cells, retinal function is unaltered in the absence of HELLS. Despite the apparent dispensable role of HELLS in retinal development, here we present evidence that loss of HELLS significantly decreases the incidence of retinoblastoma, delays tumor progression, and increases overall survival.

## Results

### *Hells* is expressed during retinal development, and repressed following terminal differentiation

We first investigated the normal expression pattern of HELLS in developing retinae. Real-time reverse transcriptase PCR (RT-qPCR) analysis of retinal tissue at different developmental stages showed that *Hells* mRNA is robustly expressed starting at early stages of retinal development (E15.5), reaching its maximal expression around postnatal day 0 (P0) and then gradually declining until it reaches its minimal expression after P6, and is maintained at low levels thereafter (Fig. 1b). The mRNA levels correlate with HELLS protein expression, with high expression of HELLS protein during the proliferative stages of retinal development (E15.5-P2), followed by a steep decrease that falls under the limits of detection after retinal cell-fate specification has been completed at P6 (Fig. 1c, d). The expression pattern of HELLS in the developing retina suggests that HELLS is expressed from an early stage of retinogenesis and likely required during cell-fate specification and differentiation.

### *Hells* is nonessential for normal retinal development

**Fig. 2:**
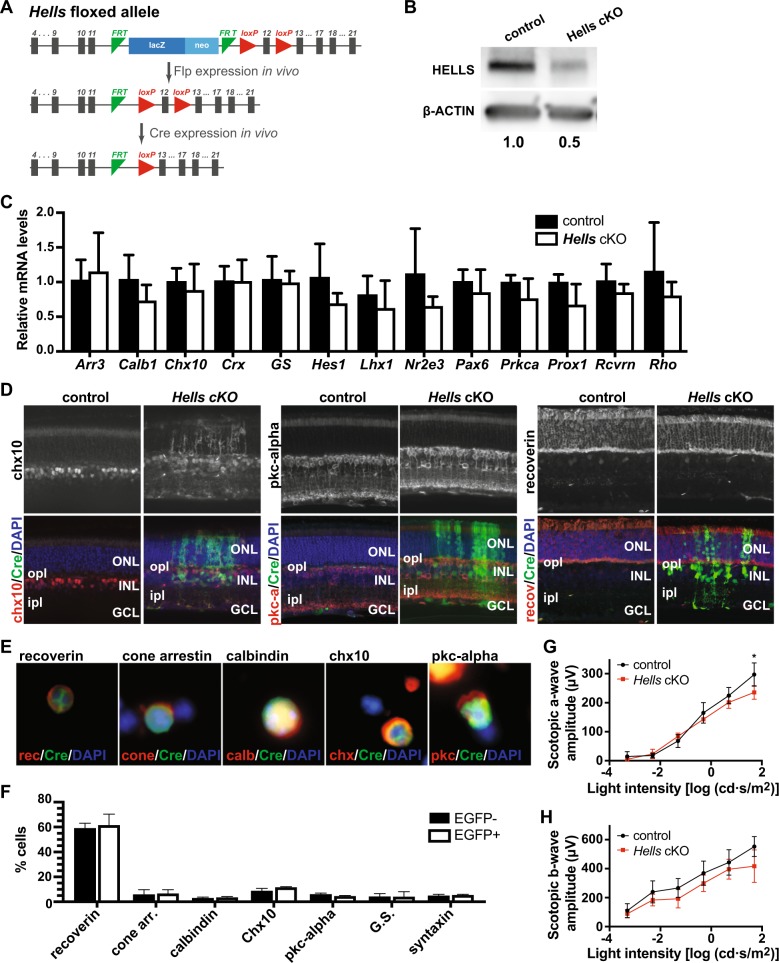
*Hells* is nonessential for normal retinal development. **a** Schematic diagram of flippase (Flp) removal of the neo-cassette and conditional excision of the floxed HELLS allele. **b** Western blot analysis of HELLS expression in *Hells* cKO (*Chx10-Cre* Hells^lox/lox^) and littermate control (Hells^lox/lox^) shows effective reduction of HELLS protein upon Cre recombination of floxed HELLS alleles. Actin was used as a loading control. Band intensities were quantified by densitometry and normalized to littermate control. **c** RT-qPCR analysis of retinal cell marker genes. The genes of the seven major retina cell types are proportionately expressed in EGFP+ cells from *Hells* cKO mice compared with cells from Cre-negative littermate controls. All data are mean ± SD normalized to control littermates (*n* = 5). **d** Representative images of P21 retina cross-sections from *Hells* cKO *Z/EG* and littermate control mice immunostained with chx10 (bipolar), pkc-alpha (bipolar), and recoverin (photoreceptors) antibodies (red). Retinae were double immunostained with anti-EGFP to capture areas of *Chx10-Cre*-mediated EGFP expression. Nuclei were counterstained with DAPI (blue). ONL outer nuclear layer, INL inner nuclear layer, GCL ganglion cell layer, ipl inner plexiform layer, opl outer plexiform layer. **e** Representative images of P21 retina dissociated cells from *Hells* cKO *Z/EG* mice immunostained with recoverin (photoreceptors), cone arrestin (cone photoreceptors), calbindin (horizontal and a subset of amacrine cells), chx10 (bipolar), and pkc-alpha (bipolar) antibodies (red). Cells were double immunostained with anti-EGFP to capture areas of *Chx10-Cre*-mediated EGFP expression. Nuclei were counterstained with DAPI (blue). **f** Quantification of the proportion of immunoreactive cells for each cellular marker antibody shown in E and Supplementary Fig. 3 was determined for EGFP+ and EGFP− cells from *Hells* cKO *Z/EG* mice from independent litters (*n* = 3). Each bar represents the mean ± SD of 500 cells scored from each retina. **g**, **h** ERGs were recorded from 5-week-old *Hells* cKO (red line) and littermate control (black line). a-wave amplitude (**g**) and b-wave amplitude (**h**) were recorded at various light intensities. All measurements are mean ± SD (*n* = 4).

To elucidate the function of HELLS during retinal development, we generated genetically engineered mice to ablate the *Hells* gene (Fig. 2a) in early retinal progenitors using the *Chx10-Cre* allele (*Chx10-Cre; Hells*^*lox/lox*^; referred hereafter as *Hells* cKO). The *Hells* cKO mouse model used in this study targets exon 12, which contains the conserved helicase domain IV. Since *Chx10-Cre* is expressed in a mosaic pattern in progenitor cells, we generated mice that also carry the *Z/EG* transgene to serve as a reporter for Cre recombination. Retinal cells that arise from the early progenitor cells that stochastically expressed Cre recombinase during early development will express enhanced green fluorescent protein (EGFP). Western blot analysis using P0 retinae, the postnatal stage with highest HELLS expression in wild-type retinae, confirmed a significant twofold reduction in HELLS protein levels in retinae expressing *Chx10-Cre* compared with *Cre*-negative littermate controls (Fig. 2b). A short form of HELLS protein was not observed using a rabbit antibody against the N-terminal sequence of HELLS, suggesting that truncated HELLS protein is not stable or below limit of detection. As mentioned, *Chx10-Cre* is expressed stochastically, therefore complete abrogation of protein expression was not expected when using whole retinae.

RT-qPCR analysis of the expression of retinal cell markers specific for the seven major retinal cell types using flow cytometry sorted EGFP + retinal cells from P21 retinae showed no significant differences in the expression of *Arr3*, *Calb1*, *Chx10*, *Crx*, *GS*, *Hes1*, *Lhx1*, *Nr2e3*, *Pax6*, *Prkca*, *Prox1*, *Rcvrn*, and *Rho* in *Hells* cKO retinae compared with *Cre*-negative littermate controls, suggesting normal retinal differentiation in the absence of HELLS (Fig. 2c).

Further evaluation of potential alterations in the development of the retina upon loss of HELLS was achieved by examining retinae cross-sections using immunohistochemistry (Fig. 2d; Supplementary Fig. 1) and immunocytochemistry of dissociated retinae (Fig. 2e; Supplementary Figs. 2 and 3). We performed immunostaining using seven different specific antibodies against each major retinal cell type. *Hells* cKO displayed no major alterations in retinal cell birthing and cell-fate specification as evidenced by the uniform layer and thickness of the outer nuclear layer (ONL), inner nuclear layer (INL), and ganglion cell layer (GCL) (Fig. 2d; Supplementary Fig. 1). All cells are located in their corresponding retinal layer. The only abnormality observed was found in a subset of bipolar cells (Chx10-immunopositive cells). We observed bipolar cellular processes extending through the outer plexiform layer (OPL) into the ONL. In comparison, the bipolar cell bodies in *Cre*-negative littermate controls and in the *Cre*-negative areas of the same retina are limited to the INL (Fig. 2d). No differences in spatial localization were observed for any of the other retinal cell types analyzed, including pkc-alpha-immunoreactive rod-bipolar cells^24^ and recoverin-immunoreactive photoreceptor cells (Fig. 2d; Supplementary Fig. 1). Immunocytochemical analysis of dissociated retinae was able to detect double-immunostained cells for every retinal cell-type-specific antibody and the EGFP reporter of Cre recombination (Fig. 2e; Supplementary Figs. 2 and 3). Quantification of EGFP + and EGFP− cell types co-stained with retinal cell markers indicated that all cells are present in the correct ratios (Fig. 2f). This confirms that all retinal cell types that are born, can terminally differentiate and survive in the absence of HELLS.

In order to determine if the aberrant bipolar processes observed in *Hells* cKO mice affect normal retinal function, we performed electroretinography (ERG) on dark-adapted *Hells* cKO mice with retinae that had at least 50% EGFP + penetrance compared with *Cre*-negative littermate controls (Fig. 2g, h). ERG measures the electrical activity of the various cell types in the retina in response to light stimulation. The a-wave hyperpolarization measures photoreceptor (cone and rod) function and the b-wave depolarization measures the inner retinal function, predominantly Müller and bipolar cells. Although *Hells* cKO mice tend to show a lower a-wave and b-wave amplitude, ERG recordings show neither significant changes in the amplitude of the a-wave, except at the highest light intensity (Fig. 2g), nor the amplitude of the b-wave (Fig. 2h), consistent with the observation that photoreceptors and inner neuron cells are comparable in numbers between *Hells* cKO and littermate controls.

### *Hells* is transcriptionally repressed by the Rb family

**Fig. 3:**
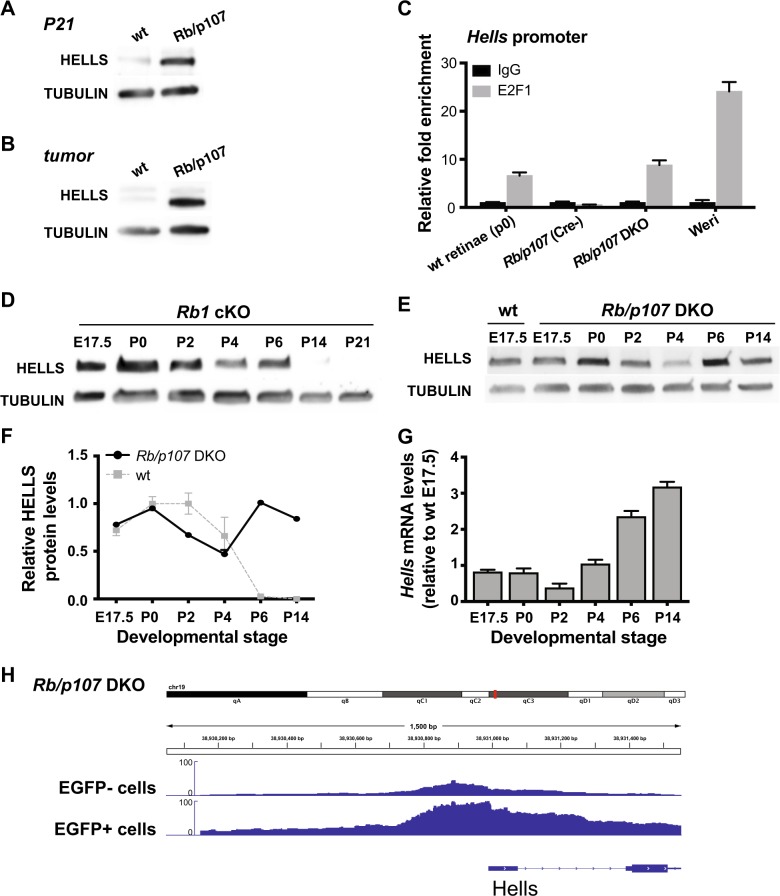
Hells is transcriptionally repressed by RB and p107. **a**, **b** HELLS protein level in mouse (**a**) P21 retinae and (**b**) retinoblastoma tumor samples were compared with littermates’ controls (wt). Tubulin was used as a loading control. **c** Chromatin immunoprecipitation (ChIP) assay in P0 and P21 *Rb1/p107* DKO mouse retinae and Weri retinoblastoma human cell line reveals enrichment of E2F1 within the *HELLS* promoter. IgG pull-down was used as negative control. **d**, **e** Western blot analysis of HELLS protein level at different retinal development stages in *Rb1* cKO mice and *Rb1/p107* DKO. Tubulin was used as a loading control. **f** Quantification of HELLS protein expression in *Rb1/p107* DKO (black line) compared to wt (gray) retinae. **g** RT-qPCR analysis of *Hells* mRNA level in *Rb1/p107* DKO retinae relative to E17.5 wild-type (wt) retinae. **h** Representative sequencing tracks for the *Hells* locus show increased peaks at the promoter in P21 *Rb1/*p107 DKO mouse retinal cells (EGFP+) compared with control retinal cells (EGFP−). The ATAC-Seq data have been normalized to take sequencing depth into account. All measurements are mean ± SD (*n* = 3).

Previous studies have shown that *HELLS* is regulated through the RB/E2F pathway and that loss of *RB1* results in upregulation and overexpression of HELLS, a process that may contribute to epigenetic changes that drive tumor progression^7,8,10^. We verified that HELLS overexpression occurs soon after tumor initiation in *Chx10-Cre Rb1*^lox/lox^ p107^−/^^−^ (*Rb1/p107* DKO; Fig. 3a) retinae and this overexpression persists in retinoblastoma tumors (Fig. 3b). To confirm that the RB/E2F pathway directly controls *Hells* transcription in the retina, we performed chromatin immunoprecipitation (ChIP) analysis using P0 wild-type and P21 *Rb1/p107* DKO and Cre-negative littermate control mouse retinae, as well as human retinoblastoma tumor cells (Weri). Pull-down using an E2F1 antibody showed enrichment of E2F1 at a consensus-binding site within the *Hells* promoter in samples that highly express HELLS protein: P0 wild-type and P21 *Rb1/p107* DKO retinae and human tumor cells, but not in P21 Cre-negative *Rb1/p107* (Fig. 3c).

To determine whether HELLS is transcriptionally repressed specifically by RB or also by other members of the RB family, we analyzed HELLS expression in *Chx10-Cre Rb1*^lox/lox^ (*Rb1* cKO) and *Rb1/p107* DKO retinae. Interestingly, we found that in *Rb1* cKO retinae, a mouse model that does not develop retinoblastoma due to compensation by p107, HELLS mRNA and protein expression follows a similar dynamic as wild-type retinae, with maximal expression during embryonic stages (E17.5-P0), repression after postnatal day 2 (P2) and significant reduction in protein levels after postnatal day 6 (P6; Fig. 3d). On the other hand, analysis of *Rb1/p107* DKO retinae at different stages of retinal development, alongside wild-type littermate controls indicate an absence of HELLS repression in *Rb1/p107* DKO mice after P4, observed both at protein (Fig. 3e, f) and transcriptional (Fig. 3g) levels. Analysis of chromatin accessibility at the *Hells* locus using ATAC-seq of P21 retinae confirmed a significant increase in reads at the *Hells* promoter and along the first two exons in EGFP-positive (*Rb*^*−/−*^*/p107*^−*/*−^*)* compared with EGFP-negative (*p107*^*−/−*^) flow-sorted *Rb/p107* DKO retinal cells (Fig. 3h). Since HELLS expression levels in *Rb1/p107* DKO retinae are comparable with those observed in wild-type retinae between E17.5 and P2 and chromatin remains open in EGFP + *Rb/p107* DKO P21 retinal cells, these results suggest that RB-family members are required for *Hells* repression during terminal differentiation of one or several retinal cell fates to block access of E2F1 at the *Hells* promoter.

### Loss of *Hells* reduces tumorigenesis in a genetic mouse model of retinoblastoma

**Fig. 4:**
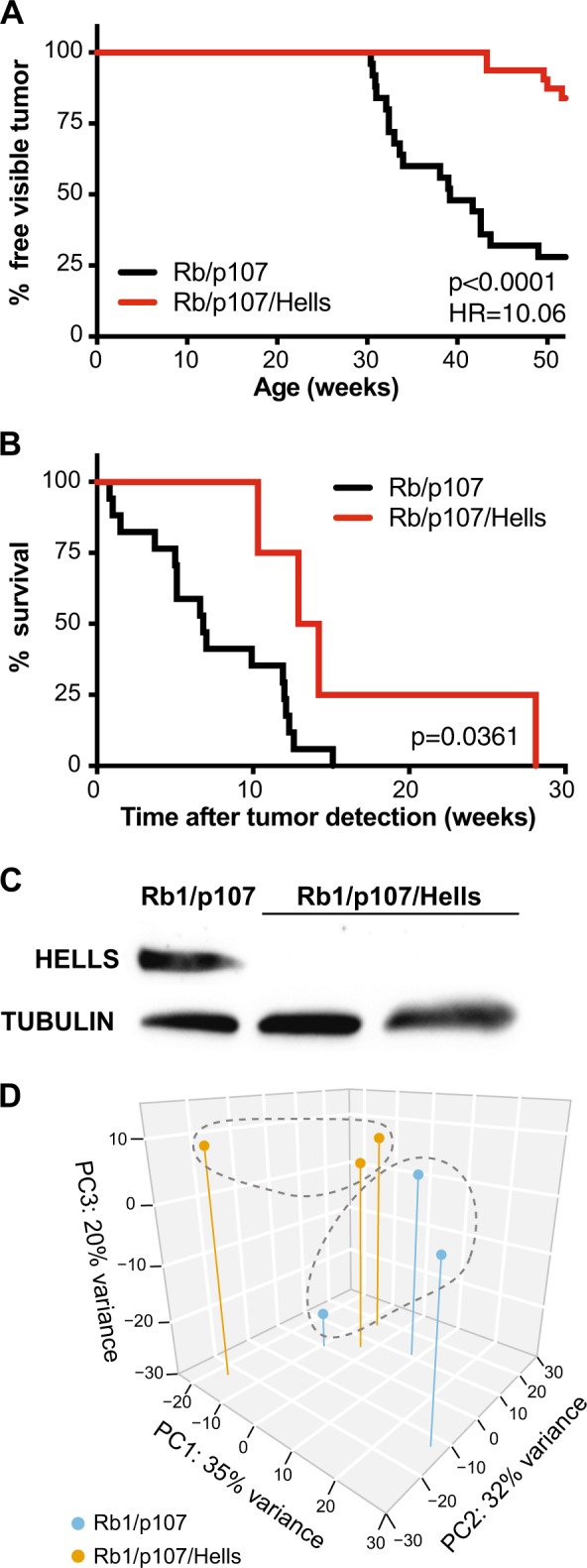
Loss of *Hells* decreases morbidity and increased survival in retinoblastoma. **a** Kaplan–Meier curves showing (**a**) the percentage of mice free of visible retinoblastoma tumors in *Rb1/p107* DKO (*n* = 18) and *Rb1/p107/Hells* (*n* = 25) and (**b**) the time to moribund status after tumor detection of retinoblastoma-bearing animals mice in *Rb1/p107* DKO (*n* = 13) and *Rb1/p107/Hells* (*n* = 4). Mantel–Cox test was used for curve comparisons. **c** Western blot detection of HELLS confirms loss of HELLS protein in *Rb1/p107/Hells* TKO tumors and HELLS expression in *Rb1/p107* DKO tumors. Tubulin used as a loading control. **d** Principal components analysis (PCA) (3D loading plot) of RNA-seq data from three *Rb/p107* DKO and three *Rb1/p107/Hells* TKO tumors show the absence of distinct transcriptome clusters.

A previous study in retinoblastoma identified HELLS as a key target gene that promotes tumor proliferation^10^. To determine if HELLS overexpression is critical for retinoblastoma development, we generated *Chx10-Cre Rb*^*lox/lox*^ *p107*^*−/−*^ *Hells*^*lox/lox*^ triple knockout (*Rb1/p107/Hells* TKO) mice and compared them to *Rb1/p107* DKO littermate controls. *Rb1/p107/Hells* TKO and *Rb1/p107* DKO mice were followed for 1 year beginning at birth for signs of tumor development. We found that abrogation of *Hells* led to a significant increase in survival and decreased morbidity in *Rb1/p107/Hells* TKO mice compared with their *Rb1/p107* DKO littermate controls (Fig. 4a). We observed that only 16% of *Rb1/p107/Hells* TKO mice (*n* = 25) developed tumors compared with 72% of *Rb1/p107* DKO mice (*n* = 18; *p* < 0.0001) in the span of 1 year (Fig. 4a). We also found that once tumors were detected, the time to moribund status for *Rb1/p107/Hells* TKO mice was on average 13.6 weeks compared to 6.8 weeks in *Rb1/p107* DKO mice (*p* = 0.0361; Fig. 4b). This translates to a significant increase of ~16 weeks in the mean survival of tumor-bearing mice from 33.8 weeks for *Rb1/*p107 DKO to 49.6 weeks for *Rb1/p107/Hells* TKO (*p* = 0.0052). We confirmed that tumors arising from *Rb1/p107/Hells* TKO mice had effectively recombined the conditional *Hells* alleles using western blot analysis (Fig. 4c).

Tumors from *Rb1/p107* DKO and *Rb1/p107/Hells* TKO mice were analyzed for gene expression using RNA-seq (*n* = 3 each). Surprisingly, despite the role of HELLS in heterochromatin formation, the gene expression profile from *Hells*-null tumors were virtually indistinguishable from controls, as portrayed by the PCA plot (Fig. 4d). Comparative transcriptome analysis of tumors from *Rb1/p107* DKO and *Rb1/p107/Hells* TKO mice rendered only 17 significant differential expressed genes (DEGs; Supplementary Fig. 4). None of the DEGs provided insightful information regarding the mechanism by which *Hells* loss reduced the incidence of retinoblastoma or improved survival.

### *Hells* loss decreases cellular proliferation in Rb1/p107-deficient retinae

Since *Rb1/p107/Hells* TKO mice develop tumors at a reduced frequency and with slower progression rates than *Rb1/p107* DKO mice, we aimed to identify pathways regulated by HELLS that aid in the delayed tumor progression. Since transcriptome analysis from tumors provided limited insight, we also performed RNA-seq and ATAC-seq analyses of P21 retinae from *Rb1/p107* DKO and *Rb1/p107/Hells* mice (*n* = 3 each) to determine if early transcriptomic differences could deepen our understanding of the mechanism through which *Hells* loss contributes to delayed tumorigenesis.

**Fig. 5:**
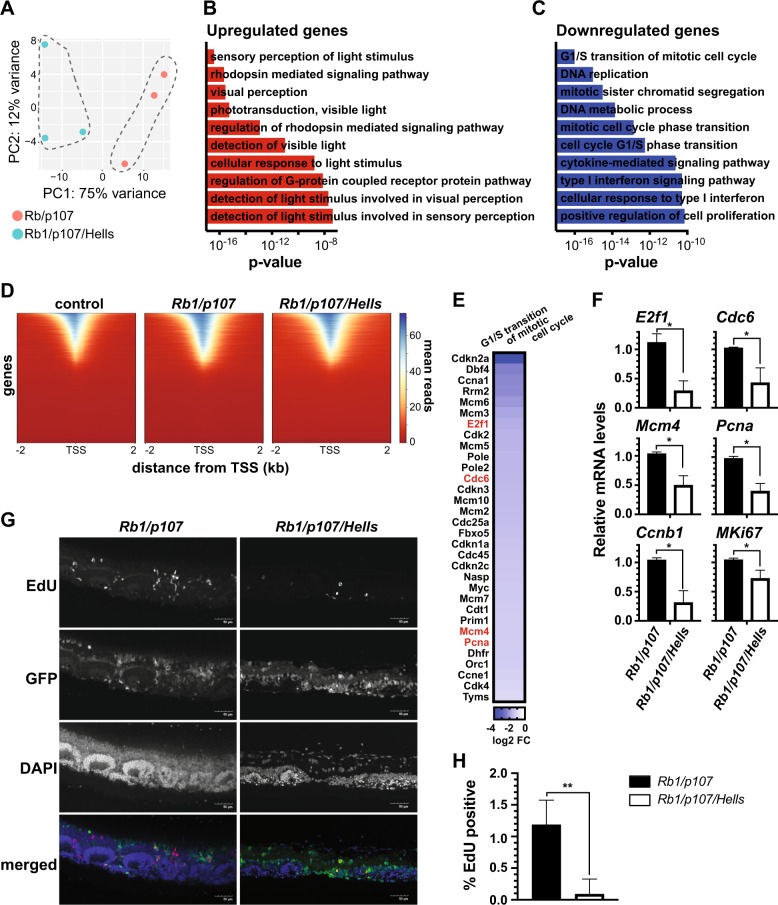
HELLS is necessary for ectopic proliferation in *Rb1/p107*-null retinae. **a** Principal components analysis (PCA) (2D loading plot) of RNA-seq data from three *Rb1/p107* DKO and three *Rb1/p107/Hells* TKO P21 retinae show distinct transcriptome clusters. **b**, **c** *p* value ranking bar graph representing the ten most significant gene ontology (GO) biological processes for (**b**) upregulated genes and (**c**) downregulated genes in *Rb1/p107/Hells* TKO compared with *Rb1/p107* DKO P21 retinae. **d** Genome-wide heatmap plot of chromatin accessibility peaks (ATAC-seq) from flow-sorted EGFP-negative Cre-negative *Rb1/p107/Hells* and EGFP-positive *Rb1/p107* DKO and *Rb1/p107/Hells* TKO P21 retina grouped by mean reads and by distance from the transcription starting site (TSS). Each row represents a gene ordered in descending accessibility mean reads. **e** Heatmap of all differentially expressed genes GO clustered as G1/S transition of mitotic cell cycle in P21 retinae from *Rb/p107* DKO compared with *Rb1/p107/Hells* TKO mice. Highlighted in red are known genes transcriptionally co-activated by HELLS. **f** Real-time RT-qPCR validation of some of the RNA-seq downregulated genes involved in cell cycle progression. *n* = 3; *p < 0.05 by unpaired *t* test. **g** Representative images of P21 retina cross-sections from *Rb1/p107* DKO and *Rb1/p107/Hells* TKO *Z/EG* mice labeled with Click-iT EdU (proliferating cells; red). Nuclei were counterstained with DAPI (blue). EGFP fluorescence by the Z/EG reporter transgene captured areas of *Chx10-Cre*-mediated EGFP expression (green). Images taken on ×20 power field. Scale bar = 50 μm. **h** Quantification of the proportion of EdU-positive cells from dissociated retinae from *Rb1/p107* DKO and *Rb1/p107/Hells* TKO mice from independent litters (*n* = 3). Each bar represents the mean ± SD of 2000 cells scored from each retina. **p < 0.0021 by unpaired *t* test.

Unlike what we found in tumors, at P21 the transcriptome profile of *Rb1/p107/Hells* TKO retinae was markedly distinctive from *Rb1/p107* DKO retinae (Fig. 5a). None of the 17 genes identified from the tumor DEG analysis (Supplementary Fig. 4) were differentially expressed in the same direction in the P21 retinae DEG analysis (data not shown). We identified a total of 1455 DEGs, with 434 upregulated genes and 1021 downregulated genes in *Rb1/p107/Hells* TKO compared with *Rb1/p107* DKO P21 retinae. Gene ontology (GO) analysis for biological processes of the 434 upregulated genes show an enrichment of genes involved in visual system-related signaling pathways and functions, as seen by the classification of the top ten most significant GO terms (Fig. 5b). Among the 1021 downregulated genes, we observed enrichment of pathways involved in DNA replication and cell division (Fig. 5c).

Since HELLS has a known role in heterochromatin formation, we performed genome-wide analysis of chromatin accessibility to determine which genes were differentially expressed due to chromatin changes following loss of *Hells*. For this, we used ATAC-seq to analyze EGFP + flow-sorted *Rb1/p107/Hells* TKO and *Rb1/p107* DKO P21 retina cells along with EGFP- control cells. In line with our previous findings^25^, *Rb1/p107* DKO retinae displayed increased areas of chromatin accessibility compared with control cells (Fig. 5d). Interestingly, *Rb1/p107/Hells* TKO compared with *Rb1/p107* DKO retinae showed only 22 regions of the genome with significant changes in chromatin accessibility, 19 of which were chromatin repression and only 3 resulting in chromatin relaxation (Fig. 5d and data not shown). Further, analysis of global levels of DNA methylation assessed by dot blot using a 5-mC-specific antibody showed no changes in genomic 5-mC levels in *Rb1/p107/Hells* TKO compared with *Rb1/p107* DKO and *Cre*-negative control retinae (Supplementary Fig. 5). These data suggest that the transcriptional changes observed upon *Hells* loss that result in decreased tumorigenesis are likely mediated through a mechanism independent of chromatin remodeling.

Previous in vitro studies have described HELLS as a transcriptional co-activator of cell proliferation, binding directly to the promoter regions of cell cycling genes, including CDC6, MKi67, PCNA, CCNB1, MCM4, E2F1, and CCNA2^26,27^. Our RNA-seq analysis detected significant downregulation in all these cell cycle genes. Four of them, *E2f1*, *Cdc6*, *Mcm4*, and *Pcna*, are part of the G1/S transition of mitotic cell cycle cluster, our most significantly enriched GO term for downregulated genes (Fig. 5e). Using RT-qPCR, we were able to independently validate downregulation of these genes in *Rb1/p107/Hells* TKO compared with *Rb1/p107* DKO P21 retinae (Fig. 5f).

To evaluate if the transcriptional downregulation of cell cycle genes in *Rb1/p107/Hells* TKO retinae results in limited proliferation, we examined P21 retinae cross-sections using immunohistochemistry (Fig. 5g; Supplementary Fig. 6) and immunocytochemistry of dissociated retinae (Fig. 5h) from mice injected with EdU 2 h prior to collection. We observed a clear decrease in the number of EdU-positive cells found along the retinae sections in *Rb1/p107/Hells* TKO P21 retinae compared with *Rb1/p107* DKO (Fig. 5g; Supplementary Fig. 6). We quantified the extent of the decrease in proliferating cells using dissociated retinae and found that the percentage of EdU-positive cells was significantly lower in *Rb1/p107/Hells* TKO (0.076 ± 0.131%) compared with *Rb1/p107* DKO (1.22 ± 0.35%; *p* = 0.0064) retinae (Fig. 5h). Together, these data suggest that HELLS overexpression (or persistent expression) may contribute to the maintenance of a de-differentiated and proliferative state in *Rb1/p107*-null retinae that may result in retinoblastoma formation.

## Discussion

In recent years, the chromatin remodeling protein HELLS has been increasingly considered as a therapeutic target in several cancers, including gliomas and carcinomas^26,28,29^. Our own studies, including this one, indicate that HELLS might be an attractive target for retinoblastoma therapeutics^10^. However, since HELLS is critical for the survival of mice and is required for normal tissue development, including the brain^17^, understanding the function of HELLS during retinal development is relevant if ocular delivery of HELLS inhibitors were to be considered as a therapeutic option for this retinal malignancy. Fortunately, unlike a previous report showing that HELLS mutant mice exhibit impaired neural progenitor cells self-renewal and maintenance during development^17^, we show in this study that *Hells* depletion does not affect RPC division, cell-fate specification, nor retinal function. Despite the seemingly dispensable role of *Hells* in retinal development, we found that persistent expression of *Hells* in *Rb1/p107-*null retinal cells is critical for ectopic proliferation and retinoblastoma tumor progression.

### *Hells* is expressed during retinal cell-fate specification stages, but is nonessential for normal retinal development

Epigenetic regulation plays a fundamental role in the maintenance of cell identity as well as the stepwise control towards cellular differentiation. Here, we studied the role of chromatin remodeler HELLS during retinal development. We found that *Hells* mRNA is expressed in early stages of retinal development and declines around postnatal day 6. We confirmed that this reduction in gene expression translated into decreased protein expression. These patterns of expression suggest that HELLS is involved in the development and cell differentiation of the retina, but is not necessary for maintenance of the retina once the major cell types have developed.

We also assessed whether HELLS is critical for normal retinal development using a conditional knockout mouse model driven by *Chx10-Cre* recombinase. We found that *Hells*-null mature (P21) retina display normal retinal cell specification and differentiation compared with their littermate controls. This suggests that HELLS is either unnecessary for retinal differentiation and development or its loss of function is compensated by other chromatin remodeling proteins.

The only difference we noted in *Hells* cKO retinae was a modest abnormal lamination of bipolar cells when compared with the surrounding Cre-negative tissue and littermate controls. Bipolar cells are the last cell type to terminally differentiate in the mouse retina, around postnatal day 6 when *Hells* expression is seen to decrease in the wild-type retina. It is possible that transcriptional repression of *Hells* helps orchestrate bipolar cell migration such that when HELLS is not present, lamination of bipolar cells is slightly altered. Despite this mild developmental defect, ERG measurements of the electrical activity of the retina in response to light stimulation indicated that *Hells-*null retina function is unaltered.

### *Hells* is downregulated by Rb and p107 during late retinal differentiation

Other groups and we have identified HELLS as a transcriptional downstream target gene of E2F1 that is overexpressed in cancer and contributes to tumor progression^9,10,30^. The evidence of direct regulation of *HELLS* by the RB family was limited to in vitro studies in gliomas and osteosarcoma^7,9^. In this study, we confirmed that the RB/E2F signaling pathway directly regulates transcriptional activation of HELLS in the developing retinae. ChIP analysis revealed enrichment of E2F1 at a known binding area on the promoter region of *HELLS*^8^ in retinal tissue during early development or in the absence of *Rb1/p107*. Interestingly, loss of *Rb1*, unlike humans, is insufficient to drive tumorigenesis in the developing murine retina, nor does it exert *Hells* derepression during terminal retinal differentiation. Our results show that in the murine retinae, loss of both *Rb1* and *p107* are necessary for the persistent expression of HELLS. These data suggest that the compensation by *p107* observed in the *Rb1* cKO mouse model is sufficient to effectively repress *Hells* expression in the absence of *Rb1*, an event that likely contributes to the prevention of tumor formation in *Rb1*-null mice^31^. Since HELLS expression levels during the early stages of retinal development (E17.5 through P2) in *Rb1/p107* DKO retinae are comparable with those observed in wild-type retinae, it is likely that the RB family is required for *Hells* repression during terminal differentiation of one or several retinal cell fates and failure to do so results in the aiding of tumor progression.

### HELLS overexpression is critical for proliferation and retinoblastoma formation in Rb1/p107-null retina

In this study, we also sought to determine whether HELLS plays a critical role during retinoblastoma development. We found that HELLS abrogation during retinoblastoma development leads to a significant decrease in morbidity and increase in survival. The mechanism(s) by which HELLS contributes to tumorigenesis, however, remain unknown. One hypothesis is that HELLS overexpression leads to increased de novo DNA methylation of tumor suppressor genes, thereby decreasing the transcriptional level of tumor suppressor genes and increasing the incidence of tumor. Given the role of HELLS in facultative heterochromatin formation, we found it intriguing that we did not observe significant changes in gene transcription in *Rb1/p107/Hells* TKO tumors compared with *Rb1/p107* DKO tumors. Given the absence of a retinal phenotype upon loss of *Hells*, it is plausible that compensation by other chromatin remodelers occurs during retinal development, something that should be explored in future studies. It is also interesting that more than twice as many gene expression changes observed in *Rb1/p107/Hells* TKO compared with *Rb1/p107* DKO P21 retinae were gene downregulations and that these changes were not associated with chromatin structure variations and that global DNA methylation was unaltered upon *Hells loss*.

**Fig. 6:**
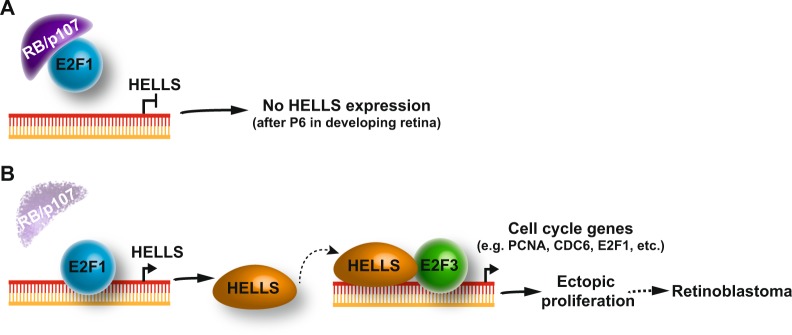
Model for HELLS function as driver of tumorigenesis in the developing retina. **a** In the normal developing retina, RB or p107 represses E2F1 from driving HELLS expression by postnatal day 6 (P6). **b** In the absence of RB and p107, E2F1 drives *Hells* transcription beyond of P6. HELLS is known to interact physically with E2F3 and function as transcriptional co-activator of E2F3. The resulting HELLS overexpression in the absence of RB and p107 mediate expression of several genes that stimulate G1/S transition and cell proliferation.

Among the most significantly downregulated transcripts in *Rb1/p107/Hells*, TKO retinae are genes involved in cell cycle regulation. As reflected by the normal functioning *Hells* cKO retina, loss of *Hells* showed no signs of alterations in the proliferation in retinal progenitor cells; however, we showed that loss of *Hells* in *Rb1/p107* differentiating retinae drastically reduced cellular proliferation. These in vivo results are in line with our previous observation in human retinoblastoma cell lines, showing reduced proliferative capacity upon *HELLS* knockdown^10^. There is a remarkable similarity between the observations in this study and studies on the E2F family. Activating E2Fs, (aE2Fs: E2F1/2/3) control the transcription of genes required for DNA replication and proliferation of quiescent cells, but are not required for normal progenitor division in several tissues, including the developing retina^32–35^. However, aE2Fs are essential for abnormal division of differentiating RB-null cells^32,34–36^ and removing E2F1 or E2F3 completely block retinoblastoma formation^10,37^. Taken together, we propose that HELLS is a critical downstream mediator of E2F-dependent ectopic division in the *Rb1/p107*-null retina (Fig. 6). In the absence of RB and p107, E2F1 transcriptional derepression drives the expression of *Hells*. HELLS then functions as transcriptional co-activator of E2F3, stimulating expression of growth promoting genes during G1/S phase transitions^27^. The resulting ectopic proliferation of differentiating retinal cells promotes retinoblastoma formation.

The decrease in tumor burden and morbidity observed in *Rb1/p107/Hells* TKO mice presents HELLS as an attractive potential therapeutic target for the treatment of retinoblastoma. This is particularly true given our observations indicating that HELLS expression is not essential for normal retinal development and that HELLS is not expressed in terminally differentiated retinal cells. This suggests that ocular delivery of HELLS inhibitors might reduce tumor burden with low retinal toxicities.

## Materials and methods

The data discussed in this publication have been deposited in NCBI’s Gene Expression Omnibus^38^ and are accessible through GEO Series accession number GSE144429.

### Mouse models

The *Rb*^*lox/lox*^ mice were obtained from the Mouse Models of Human Cancer Consortium at the National Cancer Institute; the *p107*^*−/−*^ mice were obtained from Dr. Tyler Jacks (Massachusetts Institute of Technology); *Chx10-Cre* mice were obtained from Dr. Connie Cepko (Harvard Medical School). Z/EG mice were obtained from Dr. Itsuki Ajioka (Tokyo Medical and Dental University). *Hells*^*lox/lox*^ mice were obtained from the European Mouse Mutant Archive, backcrossed to Flp mice for removal of the neo-cassette (tm1c conversion), and then backcrossed to C57BL/6N mice for final Flp removal (Fig. 2a). All mice are maintained in a C57BL/6N background, and both sexes were used in the analysis. Mice were monitored weekly, unblinded, for signs of retinoblastoma and anterior chamber invasion for 1 year from the time of birth. Moribund status was defined as the point when tumor cells invaded the anterior chamber and intraocular pressure increased to the point of imminent ocular rupture. The University of California Irvine Institutional Animal Care and Use Committee approved all animal procedures. Survival curves were generated using GraphPad Prism. Mantel–Cox test was used for statistical analysis of the Kaplan–Meier curves. Using trends from previous studies^10^, we have found that 20 mice per group were sufficient to accurately measure differences in survival with an 80% power at 0.05 significance level in *Rb1/p107/Hells* TKO compared with *Rb1/p107* DKO mice.

### Real-time reverse transcriptase PCR (RT-qPCR)

RNA was isolated from retinal tissue by homogenizing samples using Trizol Reagent and then isolated using chloroform. We used 1 µg of RNA to make cDNA according to SuperScript™III First-strand synthesis system (Invitrogen) manufacturer’s protocol at a reaction volume of 20 μl. Quantitative PCR amplification was performed using 1 µl of reverse-transcribed product in Power SYBR Green PCR Master Mix (4367659, Life Technologies). Reaction were carried out using 7500 Real-Time PCR system (Applied Biosciences). Data were normalized to endogenous 18S and GAPDH controls, and analyzed using the ΔΔ*C*_t_ method. Primers used are listed in Supplementary Table 1.

### Western blotting

Retinae were homogenized by pellet pestle in RIPA buffer (50 mM Tris-HCl, pH = 8, 150 mM NaCl, 1% NP-40, 0.5% Sodium deoxycholate, 0.1% SDS, 1 mM EDTA) with added protease inhibitor (Mini cOmplete™, Roche). Samples were placed to lyse on ice for 30 min and then centrifuged at 14,000 rpm for 30 min at 4 °C. Assessed protein concentration using the BCA protein assay (Pierce™ BCA Protein Assay Kit). In all, 30 µg of the total protein was used, and blot was run on SDS-PAGE gel (Mini-PROTEAN, Bio-rad). The gel was then transferred onto a PVDF membrane (Immobilon-P Membrane, EMD Millipore) using a semi-dry transfer apparatus (Bio-rad). The membrane was incubated in 3% nonfat dry milk dissolved in tris-buffered saline (TBS) with 0.25% Tween (TBS-T) at room temperature for an hour in order to prevent nonspecific binding. Primary antibodies were diluted in 0.5% nonfat dry milk in TBS-T as follows: 1:1000 anti-Hells (sc-28202, Santa Cruz Biotechnology), 1:2000 anti-tubulin (2144S, Cell Signaling Technology). Membranes were incubated in primary antibody overnight at 4 °C. The membranes were washed three times with TBS-T for 5 min per wash then incubated with corresponding secondary antibody for 30 min at room temperature. The secondary antibodies were prepared by diluting in 0.5% nonfat dry milk in TBS-T as follows: 1:1000 peroxidase labeled anti-mouse IgG (PI-2000, Vector Laboratories), 1:1000 peroxidase labeled anti-rabbit IgG (PI-1000, Vector Laboratories). After incubation for 30 min with secondary antibodies, the membranes were again rinsed three times with TBS-T for 5 min per wash. Bands were visualized using chemiluminescence (SuperSignal™ West Pico Chemiluminescent Substrate by Thermo Scientific). Band intensity assessed using Image J software.

### Immunohistochemistry

Retinae were isolated in phosphate-buffered saline (PBS) and fixed overnight in 4% (w/v) paraformaldehyde. Whole retinae were embedded in 4% (w/v) agarose in PBS. The retinae were then cut into 50-μm slices using a vibratome. Retinal sections were blocked in 5% (v/v) normal donkey, goat, or rabbit serum, 0.5% Triton X-100 in PBS for 4 h at room temperature and then placed in primary antibody in the same blocking solution at 4° overnight. Mouse anti-calbindin antibody (C-9848, Sigma) was used at 1:100, rabbit anti-recoverin antibody (AB5585, Millipore) was used at 1:5000 dilution, mouse anti-pH3 antibody (H6409, Sigma) was used at 1:200 dilution, mouse anti-syntaxin antibody (S0664, Sigma) was used at 1:500 dilution, mouse anti-glutamine synthetase (610518, BD Biosciences) was used at 1:100 dilution, rabbit anti-cone-arrestin antibody (AB15282, Millipore) was used at 1:5000 dilution, and sheep anti-chx10 antibody (X1180P, Exalpha Biological) was used at 1:200 dilution. The retinal sections were washed three times with PBS and incubated in corresponding secondary antibody diluted 1:500 in respective blocking buffer (goat, rabbit, or donkey) for 1 h at room temperature in the dark. Again, retinae were washed three times with PBS and then incubated in 300 μl of Vectastain ABC kit (Vector Laboratories) for 30 min at room temperature. Retina washed three times in PBS, and then placed in 300 μL tyramide Cy3 1:125 in amplification buffer (Perkin Elmer) for 10 min. Retinal sections were washed with PBS three times, and DAPI was added (1:1000 dilution in PBS) for 10 min to stain the nuclei. Retina washed two times with PBS, and mounted on slides. Imaging done using a Zeiss Confocal microscope.

### Immunocytochemistry of dissociated retina

Retina were isolated and dissociated using trypsin 100X stock and incubation at 37 °C for 10 min. Trypsin inhibitor and 1000X DNase I were added to sample and incubated at 37 °C for 5 min. Complete culture medium was then added, and cells were transferred to chamber slides. The chamber slides were prepared with 1Xpoly-L-lysine incubated for 5 min. Cells were allowed to incubate on the slides for 30 min at 37 °C. Then, media was aspirated, and cells were fixed using 4% (w/v) paraformaldehyde overnight at 4 °C. Slides washed with PBS twice. Slides were then incubated with primary antibody overnight at 4 °C. Mouse anti-calbindin antibody (C-9848, Sigma) was used at 1:100, rabbit anti-recoverin antibody (AB5585, Millipore) was used at 1:5000 dilution, mouse anti-pH3 antibody (H6409, Sigma) was used at 1:200 dilution, mouse anti-syntaxin antibody (S0664, Sigma) was used at 1:500 dilution, mouse anti-glutamine synthetase (610518, BD Biosciences) was used at 1:100 dilution, rabbit anti-cone-arrestin antibody (AB15282, Millipore) was used at 1:5000 dilution, and sheep anti-chx10 antibody (X1180P, Exalpha Biological) was used at 1:200 dilution. Slides washed three times in PBS then incubated with secondary antibody in 1:500 dilution with the corresponding blocking buffer for 30 min at room temperature in the dark. Slides were washed three times with PBS, and incubated in 300 μl of Vectastain ABC kit (Vector Laboratories) for 30 min at room temperature. Slides were washed three times in PBS and incubated with tyramide Cy3 1:150 in amplification buffer (Perkin Elmer) for 10 min. After washing with PBS three times, DAPI was added (1:1000 dilution in PBS) for 5 min to stain the nuclei. Slides were washed twice with PBS and mounted using gelvatol containing DABCO. Imaging was completed using Life Technologies EVOS microscope. Cell scoring was performed by a blinded investigator.

### Electroretinography (ERG)

For scotopic ERG measurement, mice were dark adapted for 24 h prior to ERG recording. Each group consisted of four 5-week-old mice. Under dim red light, mice were anesthetized by intraperitoneal injection of a cocktail consisting of 20 mg/ml ketamine and 5 mg/ml xylazine in phosphate-buffered saline at a dose of 0.1 ml per 20 g body weight. Pupils were dilated with 1% tropicamide (Henry Schein, Melville, NY), and applied with 2.5% hypromellose (Akorn, Lake Forest, IL) to keep corneas hydrated. Contact electrodes were placed on corneas while the reference electrode needle was positioned subdermally between the ears. The a-wave and b-wave responses were recorded followed by a white light stimulus of different flash intensities (−3.3 to 1.7 log cd·s/m^2^). For each intensity, 3–20 recordings were made with the resting intervals for recovery from photobleaching and were averaged for the final amplitude. All ERGs were recorded with the Celeris ophthalmic electrophysiology system (Diagnosys LLC, Lowell, MA) and analyzed with Espion V6 software (Diagnosys LLC). Statistical analysis was performed with paired *t* test. Data are represented as means ± SD. The investigator performing ERG measurements was blinded to the mouse genotype.

### Chromatin immunoprecipitation (ChIP assay)

ChIP assays were performed on Weri retinoblastoma human cell line, postnatal day 0 retinae collected from wild-type mice, or postnatal day 21 retinae collected from *Rb1/p107 DKO* mice as previously described^25^. The antibodies used for chromatin pull-down were anti-E2F1 (3742; Cell Signaling) and rabbit IgG (sc-2027, Santa Cruz Biotechnologies). ChIP DNA was analyzed by qPCR with SYBR Green (Bio-Rad) in ABI-7500 (Applied Biosystems). Primers used are listed in Supplementary Table 1. The primers flank areas of homology in the mouse and human promoter region locater between position −94 and 114 bp and −115 and −134 bp, respectively. In human, this sequence falls within a functionally validated E2F1-binding region^8^. ChIP experiments were run in triplicate for each of the three independent retinal samples per condition (*n* = 3).

### RNA sequencing

The total RNA was isolated using the RNA Micro Kit (Qiagen). Subsequently, 500 ng of the total RNA was used to create the RNA-seq library following the manufacturer’s protocol from purification, mRNA fragmentation through the adenylation of end-repaired cDNA fragments and cleanup (TruSeq Stranded mRNA, Illumina). The collected sample was cleaned with AMPure XP beads (Beckman Coulter) and eluted in 20 μl of 10 mM Tris buffer, pH 8, 0.1% Tween 20. A paired-end 100-bp sequencing run was performed on HiSeq 4000 yielding 348 M PE reads with a final library concentration of 2 nM as determined by qPCR (KAPA Biosystem).

Sequencing reads were aligned to the mouse reference genome (GRCm38, ENSEMBL v.92) using STAR (v2.5.2a)^39^. Each read pair was allowed a maximum number of mismatches of 10. Each read was allowed a maximum number of multiple alignments of 3. Gene counts for each sample were produced using HTSeq (v0.6.1p1)^40^. Read count normalization and differential expression analysis were performed using DESeq2 (v1.22.2) in R (v3.5.2)^41^. Genes with low reads (sum across samples <10) were removed. Differentially expressed genes (DEG) were calculated by comparing *Rb1/p107/Hells* TKO to *Rb1/p107* DKO, genes with base mean ≥10, Log2FoldChange ≥ 1 or ≤ −1, and adjusted *p* value (Benjamini–Hockberg) ≤ 0.05 were called differentially expressed genes. 3D PCA plot was generated using the R package plot3D (v1.1.1).

### Chromatin profiling

Approximately 50,000 cells were harvested for ATAC-seq for each replicate. Briefly, retinae were dissociated, flow sorted into EGFP− or EGFP + , assessed for cell viability, counted, and washed with PBS. ATAC-seq was performed as previously described^42^. ATAC-seq libraries were sequenced with the Illumina HiSeq 4000 using 100 bp paired-end single indexed run. Raw reads were first QCed (*FASTQC*) and quality and adapter trimmed using *Trimmomatic*. Trimmed reads were then aligned to mm10 build of the mouse genome using Bowtie2 (v2.2.5) with alignment parameters: bowtie2 -X 2000– local–dovetail. Potential PCR duplicate reads were removed using *MarkDuplicates* from the *Picard* tools. Peaks were called in each sample using *MACS2* and further filtered using the ENCODE consensus blacklist regions (http://mitra.stanford.edu/kundaje/akundaje/release/blacklists/mm10-mouse/). Differential peaks were identified using R package *diffbind* across the consolidated peak sets and metrics such as adjusted *p* values were reported. Heatmap plot was generated using deepTools3.

### 5-mC DNA dot blot

Genomic DNA was extracted from P21 retinae (Wizard SV genomic DNA purification Kit). Purified DNA was quantified, sonicated, denatured, and transferred to the Nylon membrane (RPN303B, GE Healthcare) according to the Cell Signaling DNA Dot Blot Protocol. 5methylcytosine (5-mC) Ab (#28692, Cell Signaling) was used to detect global DNA methylation of the samples. Methylene blue staining was used to verify equal DNA loading across samples.

## Supplementary information

Supplemental Figure Legends

Supplemental Figure 1

Supplemental Figure 2

Supplemental Figure 3

Supplemental Figure 4

Supplemental Figure 5

Supplemental Figure 6

Supplemental Table 1
