# Supplementary material for: Chromatin remodeling protein HELLS is critical for retinoblastoma tumor initiation and progression
Source: Oncogenesis. 2020 Feb 18;9(2):25. doi: 10.1038/s41389-020-0210-7 (PMC7028996; doi:10.1038/s41389-020-0210-7)
Supplement: Supplementary file 1 — Supplemental Figure Legends [file 41389_2020_210_MOESM1_ESM.docx]

**SUPPLEMENTAL FIGURE LEGENDS**

**Supplemental Figure 1**

Representative images of P21 retina cross-sections from *Hells* cKO *Z/EG* mice and their littermate controls immunostained with recoverin (photoreceptors), cone-arrestin (cone photoreceptors), calbindin (horizontal and a subset of amacrine cells), chx10 and pkc-alpha (bipolar), glutamine synthetase (G.S.; glial), and syntaxin (amacrine) antibodies (red). *Hells* cKO *Z/EG* retina were double immunostained with anti-EGFP to capture areas of *Chx10-Cre*-mediated EGFP expression. Nuclei were counterstained with DAPI (blue). ONL, outer nuclear layer; INL, inner nuclear layer; GCL ganglion cell layer; ipl, inner plexiform layer; opl, outer plexiform layer.

**Supplemental Figure 2**

Representative images of single dissociated cells from P21 retina from *Hells* cKO *Z/EG* mice that co-express EGFP and cell specific markers. The cells were immunostained with recoverin (photoreceptors), cone-arrestin (cone photoreceptors), calbindin (horizontal and a subset of amacrine cells), chx10 and pkc-alpha (bipolar), syntaxin (amacrine), and glutamine synthetase (G.S.; glial), antibodies (red). Nuclei were counterstained with DAPI (blue).

**Supplemental Figure 3**

Representative images of dissociated cells from P21 retina from *Hells* cKO *Z/EG* mice. The cells were immunostained with recoverin (photoreceptors), cone-arrestin (cone photoreceptors), calbindin (horizontal and a subset of amacrine cells), chx10 and pkc-alpha (bipolar), glutamine synthetase (G.S.; glial cells), and syntaxin (amacrine) antibodies (red). Nuclei were counterstained with DAPI (blue). Images taken on 40X power field. Scale bar = 100 μm.

**Supplemental Figure 4**

Heatmap with read counts after variance-stabilizing transformation from differentially expressed genes observed in tumors analyzed from *Rb/p107* DKO mice (n=3) compared to tumors from *Rb1/p107/Hells* TKO mice (n=3).

**Supplemental Figure 5**

Representative 5-mC-specific dot blot assay of gDNA from P21 retinae from Cre-negative *Rb1/p107/Hells* compared to *Rb1/p107* DKO and *Rb1/p107/Hells* TKO. Serially diluted genomic DNA was used to test global levels of 5mC DNA methylation. Methylene blue staining was used as loading control.

**Supplemental Figure 6**

Representative images of P21 retina cross-sections from *Rb1/p107* DKO and *Rb1/p107/Hells* TKO *Z/EG* mice labelled with Click-iT EdU (proliferating cells; red). Nuclei were counterstained with DAPI (blue). EGFP-fluorescence by the Z/EG reporter transgene captured areas of *Chx10-Cre*-mediated EGFP expression (green). Images taken on 10X power field. Scale bar = 100 μm.
