# Supplementary figures and images for: Chromatin remodeling protein HELLS is critical for retinoblastoma tumor initiation and progression
Source: Oncogenesis. 2020 Feb 18;9(2):25. doi: 10.1038/s41389-020-0210-7 (PMC7028996; doi:10.1038/s41389-020-0210-7)

*Chx10-Cre Hells<sup>lox/lox</sup>*

*Hells<sup>lox/lox</sup>*

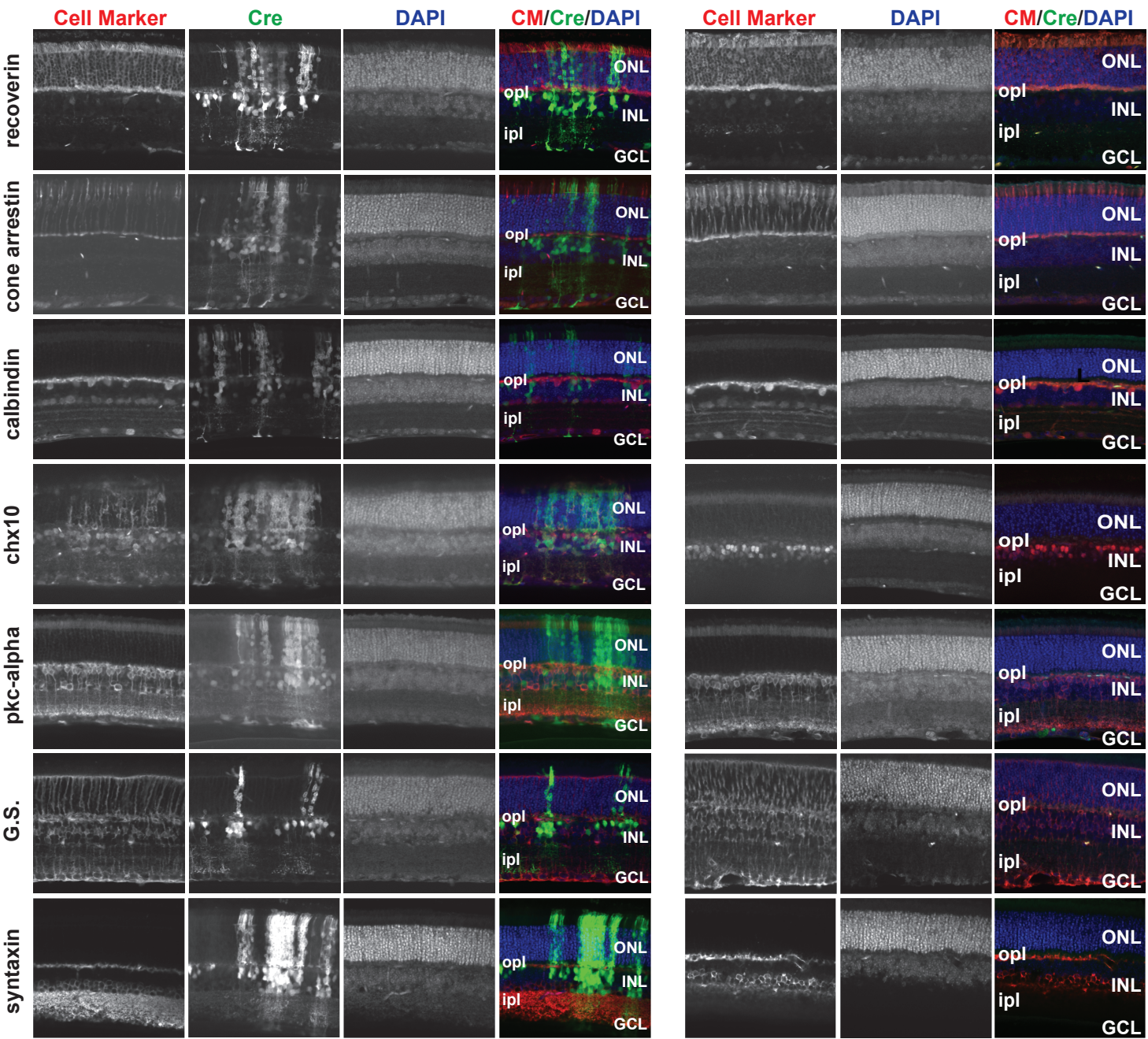

Supplement: Supplementary file 2 — Supplemental Figure 1 [file 41389_2020_210_MOESM2_ESM.pdf]

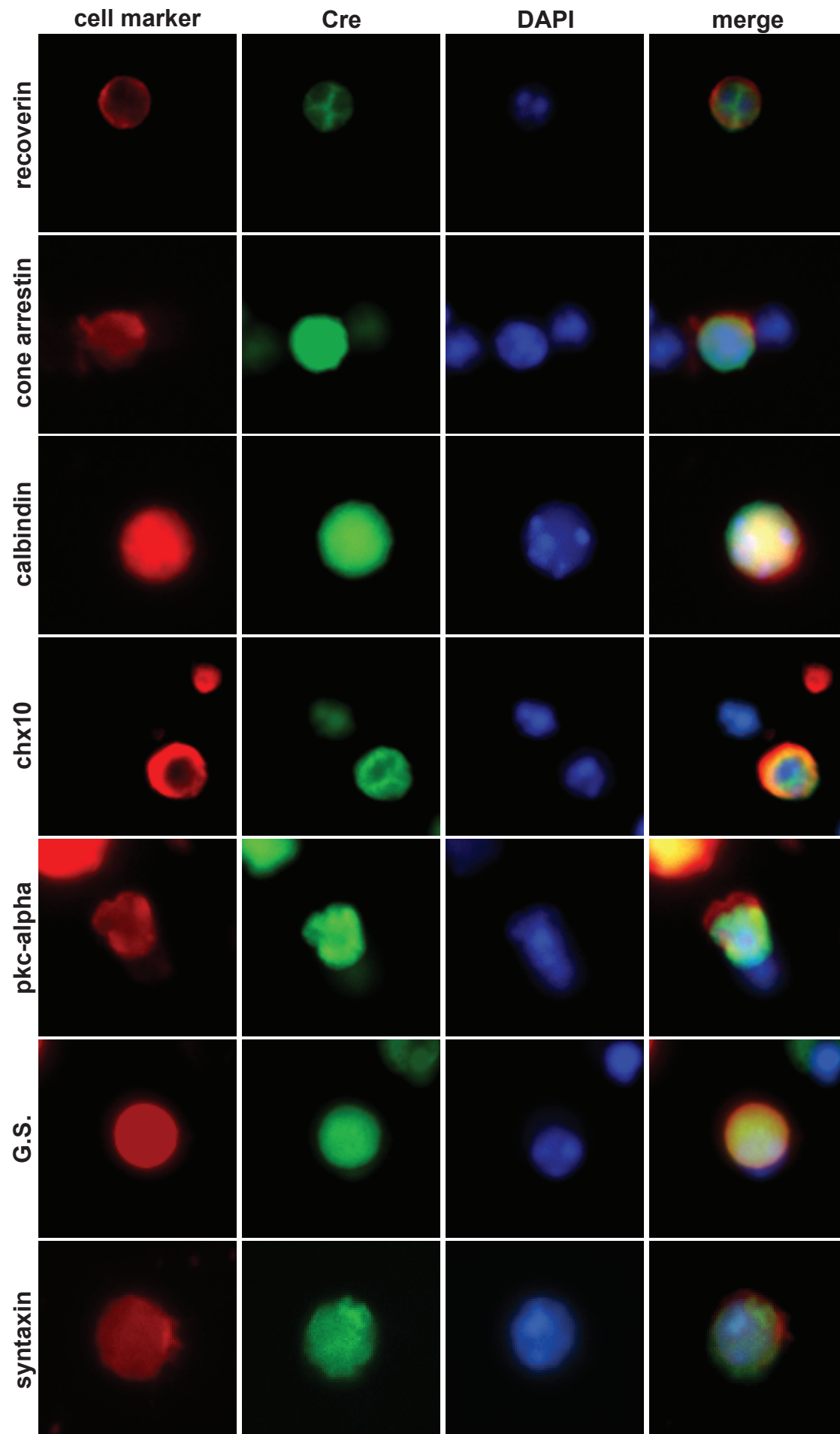

Supplement: Supplementary file 3 — Supplemental Figure 2 [file 41389_2020_210_MOESM3_ESM.pdf]

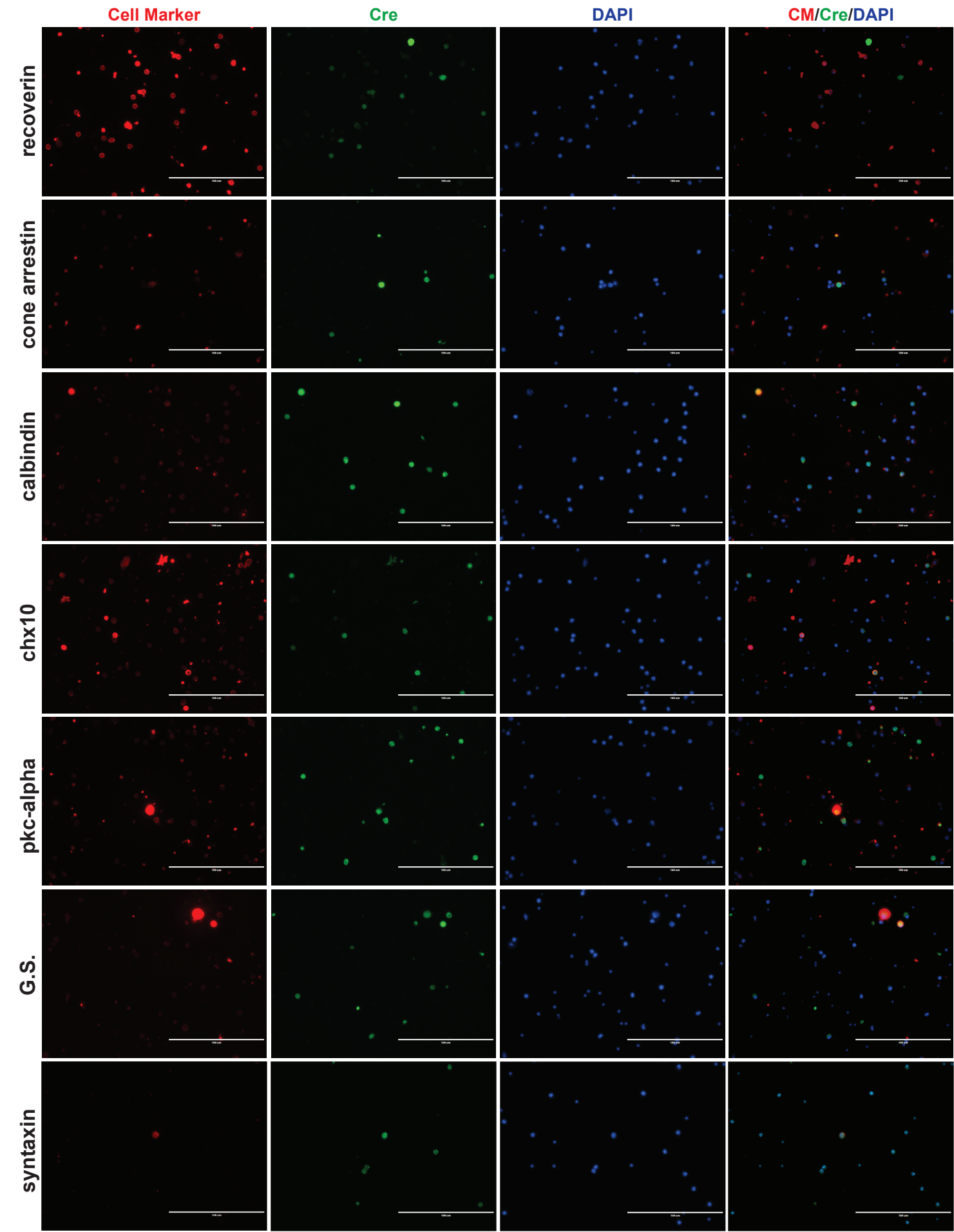

Supplement: Supplementary file 4 — Supplemental Figure 3 [file 41389_2020_210_MOESM4_ESM.pdf]

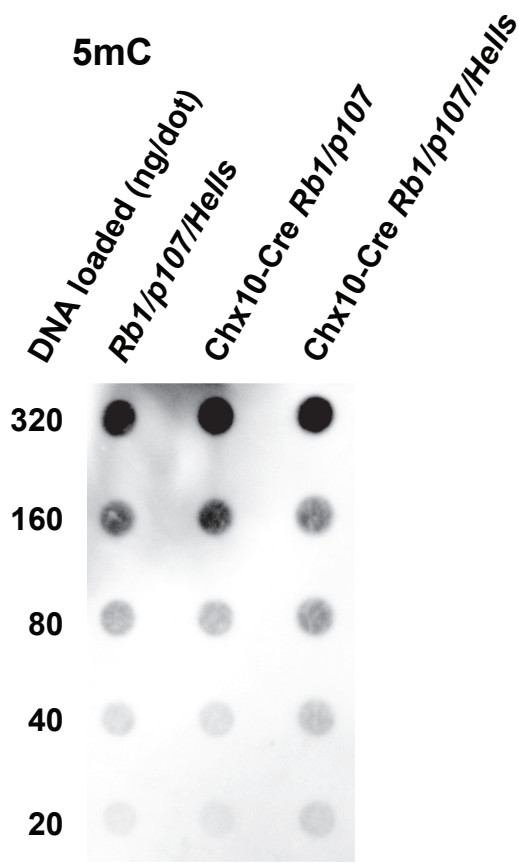

**Methylene blue**

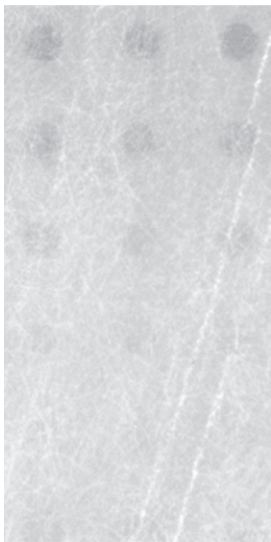

Supplement: Supplementary file 6 — Supplemental Figure 5 [file 41389_2020_210_MOESM6_ESM.pdf]

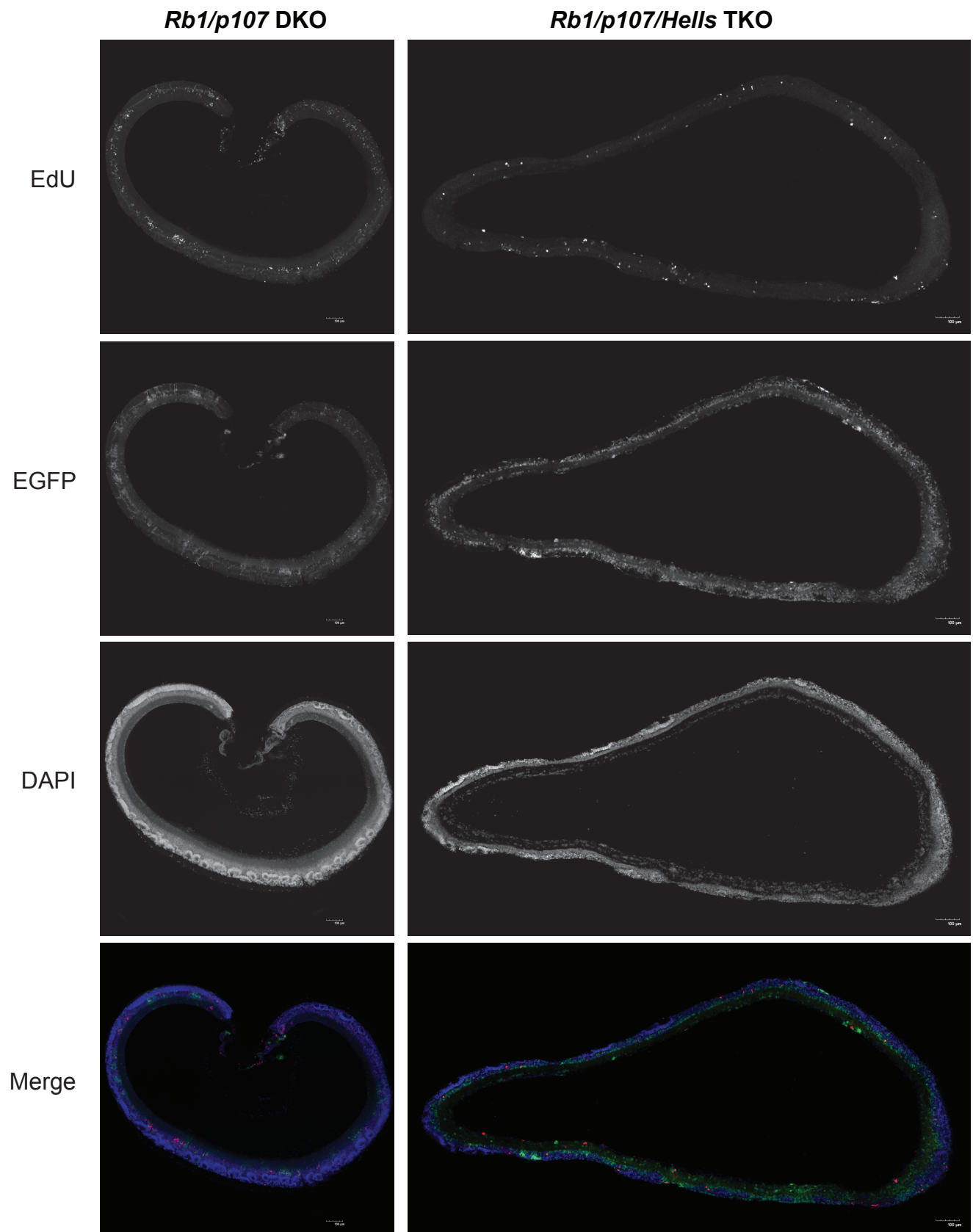

Supplement: Supplementary file 7 — Supplemental Figure 6 [file 41389_2020_210_MOESM7_ESM.pdf]
