# Supplementary material for: Chromatin remodeling protein HELLS is critical for retinoblastoma tumor initiation and progression
Source: Oncogenesis. 2020 Feb 18;9(2):25. doi: 10.1038/s41389-020-0210-7 (PMC7028996; doi:10.1038/s41389-020-0210-7)
Supplement: Supplementary file 8 — Supplemental Table 1 [file 41389_2020_210_MOESM8_ESM.pdf]

Table S1. Real-time PCR primers

**Gene**	**Forward**	**Reverse**
Arr3	TGACTTGGATGTGATTGGTCTGA	GGAGCCACTTGCTTGGTTTG
Calb1	CACATGTAACTCTGTTTCGTGTATCCTT	TCACAATAAAGAATCCAGGCAATTAA
Chx10	TGAGGCAAGGCCCATGTC	CGGGAGTATGTCCAGGATGTCT
Crx	TCTGTGTGTTACAGACATGACCACTAA	CATCAAGCTTCTTTTGCATTTTGT
GAPDH	CTCCACTCACGGCAAATTCA	CGCTCCTGGAAGATGGTGAT
Glutamine Synthetase	GGTGCCAAGTTTGAGTGATGAG	ACTTTCCCGGTACTGCATCCT
Hells	CCATTGCCAAGTTTATCAGTGTT	CTGAAGTCCAAATCCCATGAATT
Hes1	AGAGCCTCAGGCCACTGCTA	TTCGTTTTTAGTGTCCGTCAGAAG
Lhx1	GCTACGGGAACCATTTGTCTCA	GCGAGACCCCGCTACCA
Nr2e3	AGGCTGGAAGTTGAACAAAAGC	TCCAGTCTCCCTCCTTTCCC
Pax6	CACCGCCCTCACCAACAC	AGGTTGTTTGCCATGGTGAAG
Prkca	CGCGAGGACAGCCTGTCT	AGAACCCTTCAAAATCAGATTGGT
Prox1	TTAGAACAGGCTCGTGGTGAGA	TGAAGCAAAGTAAATAGCAACTAGTGACA
Rcvrn	GCAGCTTCGATGCCAACAG	TCATGTGCAGAGCAATCACGTA
Rho	AGCAGCAGGAGTCAGCCACC	CCGAAGTTGGAGCCCTGGTG
Atrx	TTATGAATAGACGGCTCCAGC	GCTGGTAGTTAGAAGGAGTCATG
Top2a	GTGGACTTGGAAGACACGATAG	GTCAGAGGTTGAGCACTGTATC
human HELLS promoter	CCTGAGAGAGGTCCAGGTAAA	CTGTCATCTCGCGATACCTTAC
mouse Hells promoter	GAACAACTCGGGACCATCATTA	CTAGCTCCAGCCTTGAATGTC
E2f1	GCTGGAGAAACAGTCCCTTTGT	ACGTACCCTCCCCATCTCAGA
Cdc6	TTTCATCTGAGCCCCTCTTTC	TGCCCCTTCCCTCAAAAC
Mcm4	AGAGCCTAAGCCCAGTTTTG	CACAGTAAGGACATAGCCTCTAC
Pcna	GGCTCCCAAGATTGAAGATGAG	GGTGACAGAAAAGACCTCAGG
Ccnb1	GTAACATAGTCATTCCCTCGGTG	GTCAGAATTCAAAGCACACCC
MKi67	GTCATGAGGAAGATCACCAGG	GCTTTATTGGATAGGACAGAGGG
